# Supplementary material for: Enhancing hydrovoltaic power generation through heat conduction effects
Source: Nat Commun. 2022 Feb 24;13:1043. doi: 10.1038/s41467-022-28689-8 (PMC8873497; doi:10.1038/s41467-022-28689-8)
Supplement: Supplementary file 1 — Supplementary Information [file 41467_2022_28689_MOESM1_ESM.pdf]

Supplementary information

Enhancing Hydrovoltaic Power Generation Through Heat Conduction Effects

Lianhui Li[1#], Sijia Feng[1#], Yuanyuan Bai[1], Xianqing Yang[1], Mengyuan Liu[1], Mingming Hao[1], Shuqi Wang[1], Yue Wu[1], Fuqin Sun[1], Zheng Liu[2], Ting Zhang[1,3,4]*

[1]*i*-Lab, Key Laboratory of multifunctional nanomaterials and smart systems, Suzhou Institute of Nano-Tech and Nano-Bionics (SINANO), Chinese Academy of Sciences (CAS). 398 Ruoshui Road, Suzhou, 215123, P. R. China.

[2]School of Materials Science and Engineering, Nanyang Technological University, Singapore, Singapore.

[3]Center for Excellence in Brain Science and Intelligence Technology, Chinese Academy of Sciences (CAS), 320 Yueyang Rod, Shanghai 200031, China.

[4]Gusu laboratory of materials, 388 Ruoshui Road, Suzhou, 215123, P. R. China.

[#] These authors contributed to this work equally.

E-mail: **tzhang2009@sinano.ac.cn**

Supplementary Figures

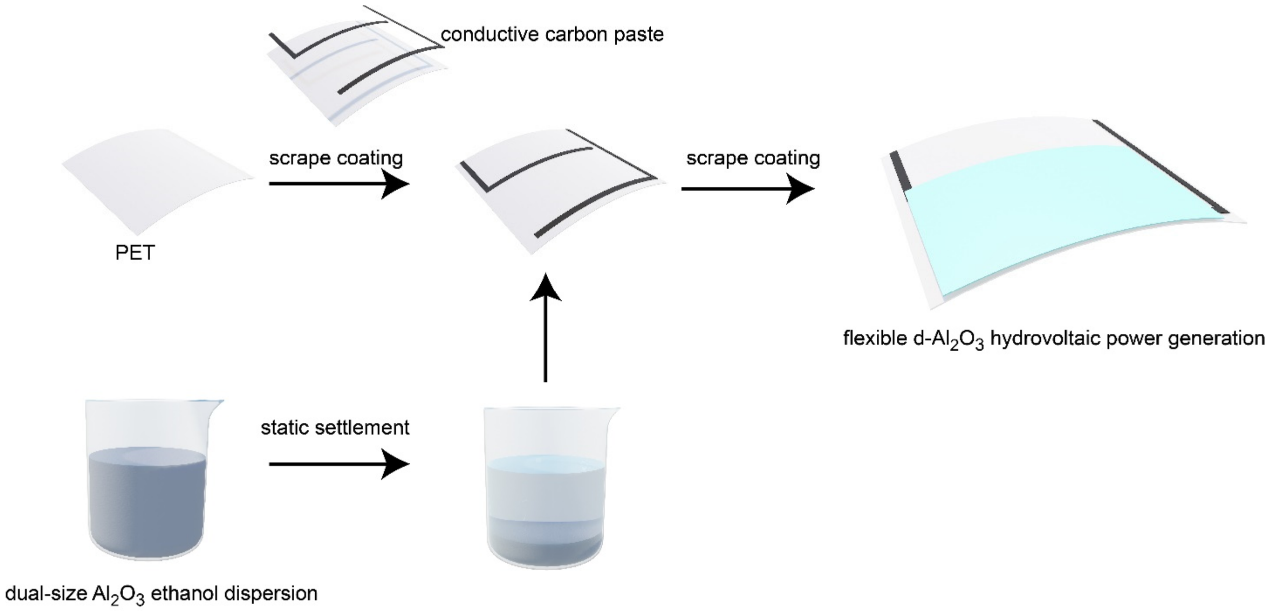

Supplementary Figure 1. Schematic illustration of the preparation process of the flexible porous *d*-Al_2O_3 hydrovoltaic generator.

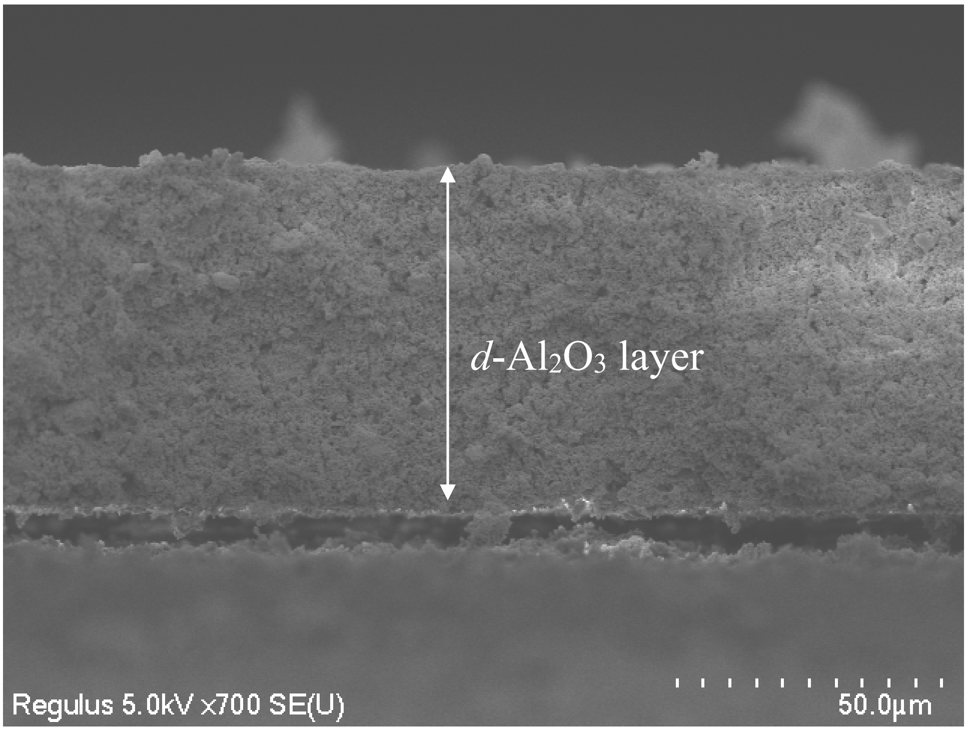

Supplementary Figure 2. Cross-sectional Scanning electron microscope (SEM) images of porous *d*-Al_2O_3 film.

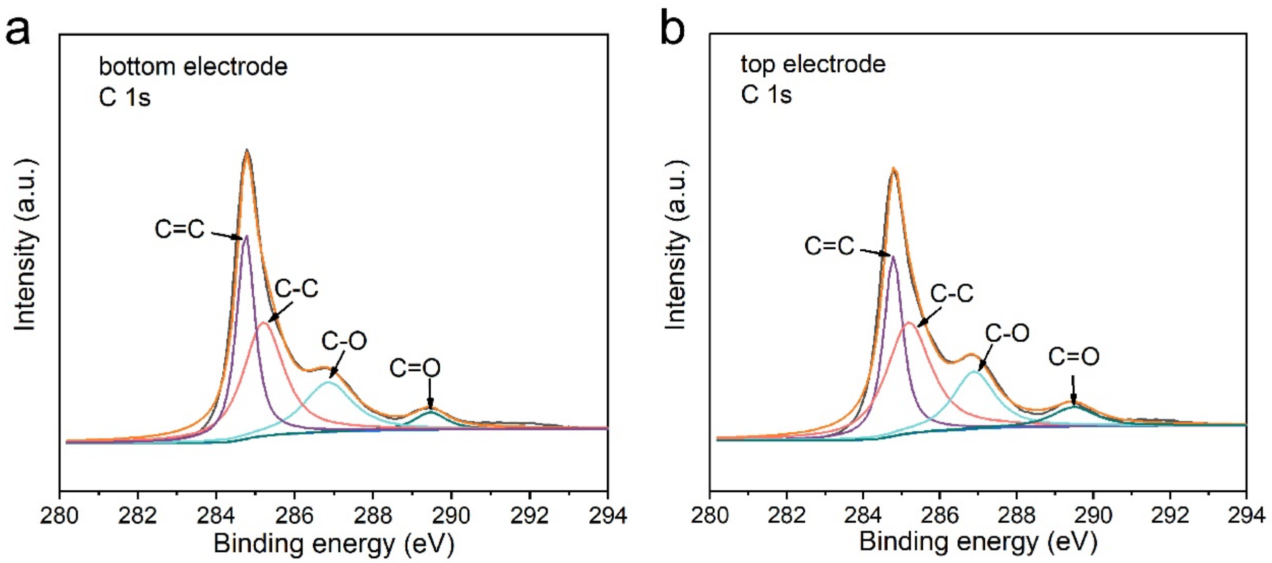

Supplementary Figure 3. high-resolution X-ray photoelectron spectroscopy (XPS) of C1s elements of the bottom (a) and top (b) electrodes.

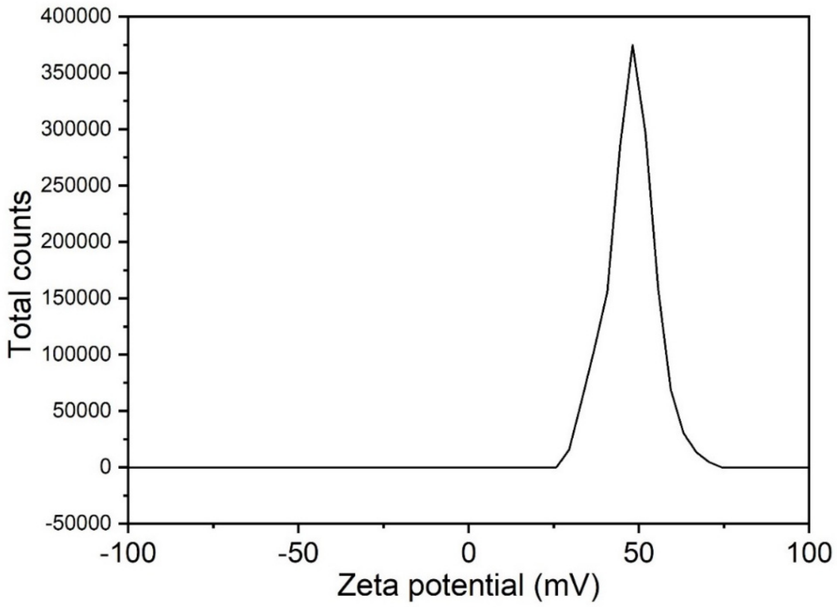

Supplementary Figure 4. Zeta potential measurement of dual-size Al_2O_3 particles in distilled water.

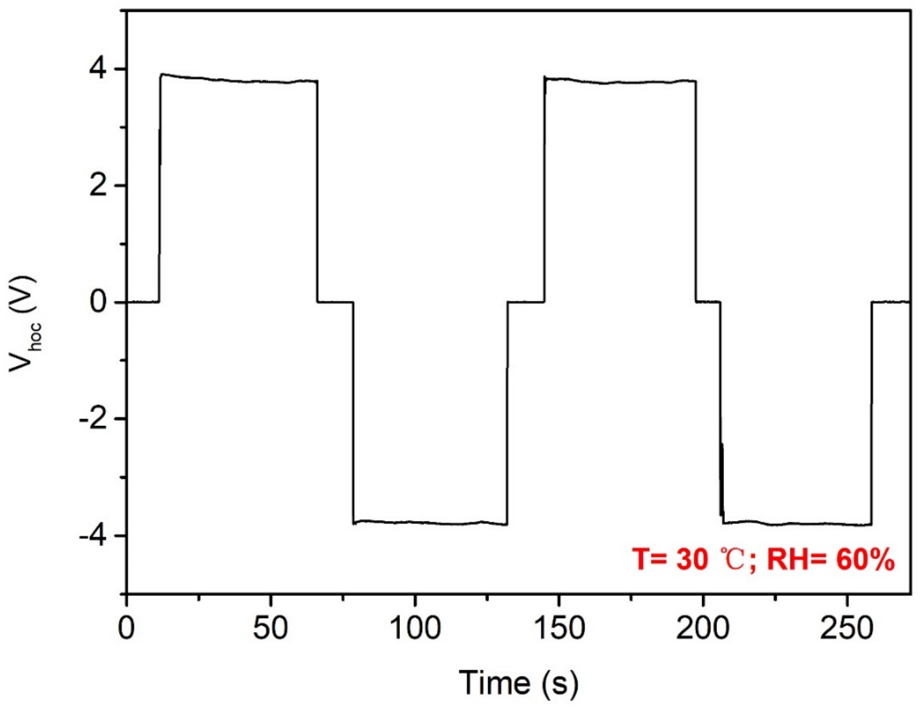

Supplementary Figure 5. Open circuit voltage response versus time curve of porous *d*-Al_2O_3 hydrovoltaic generator with switching the upper and lower electrodes.

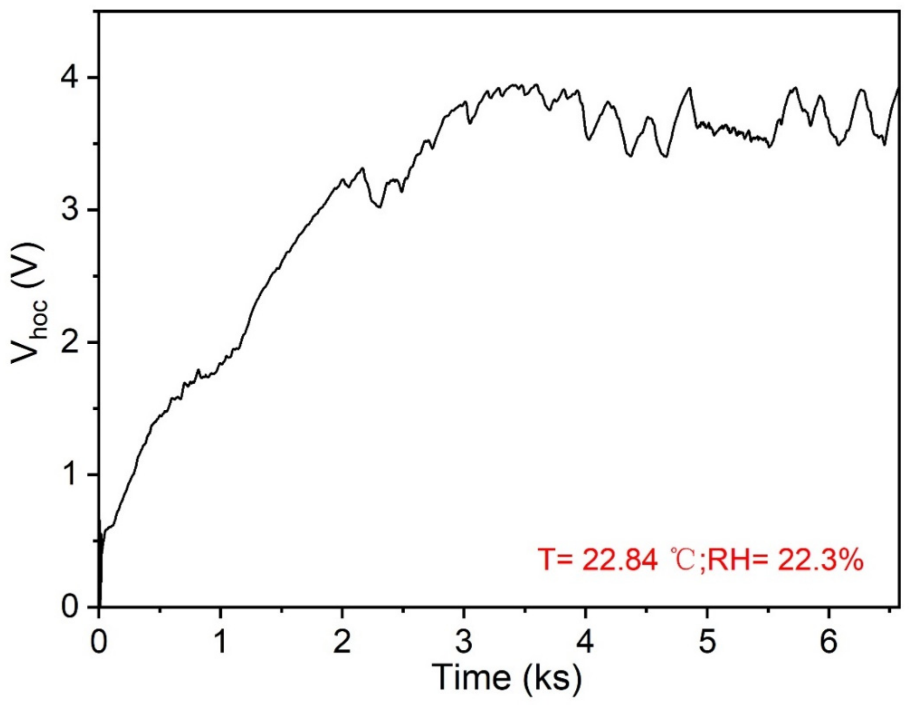

Supplementary Figure 6. Open circuit voltage response versus time curve when the bottom of porous *d*-Al_2O_3 hydrovoltaic generator is immersed in DI water at the ambient temperature of 22.8°C and humidity of 22.3% RH.

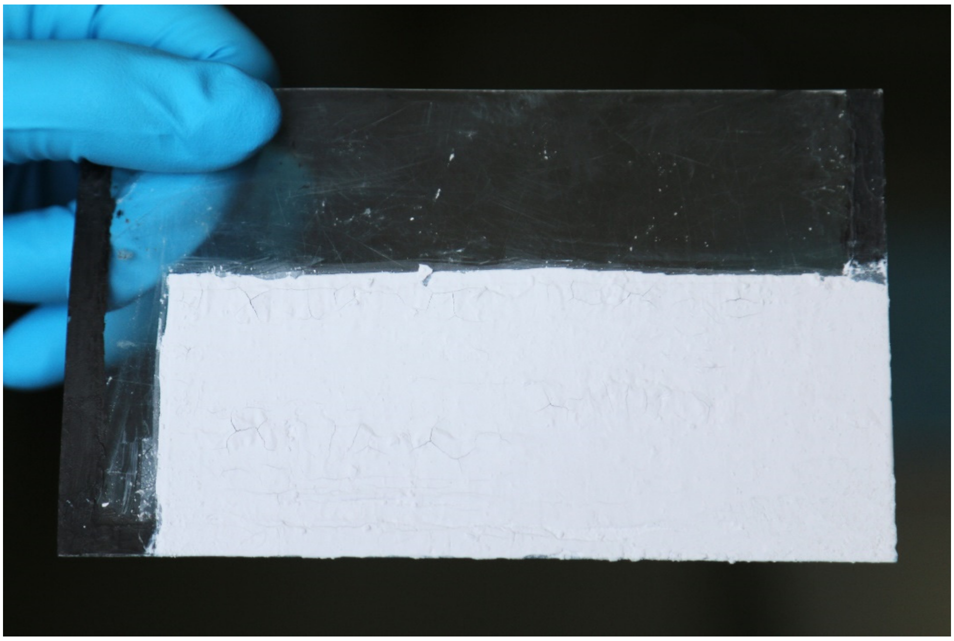

Supplementary Figure 7. Optical images showing a HG device with m_{200} : m_{20} of 5:1 *d*-Al_2O_3 layer under relaxed state.

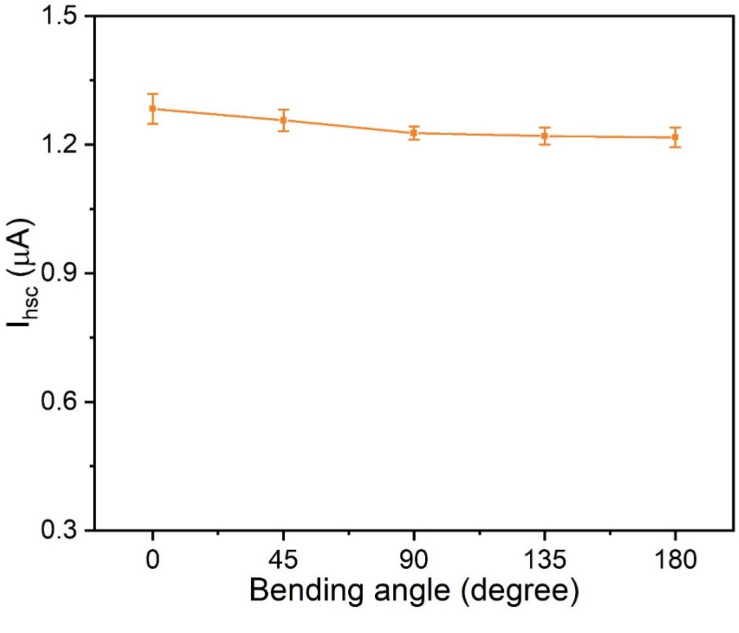

Supplementary Figure 8. Variation of short-circuit current of the device under different bending state at 295 K and 48% RH. The width of the device is 10 cm. The error bar is received from the I_{sc} of three HGs under steady operating condition in the same bending state.

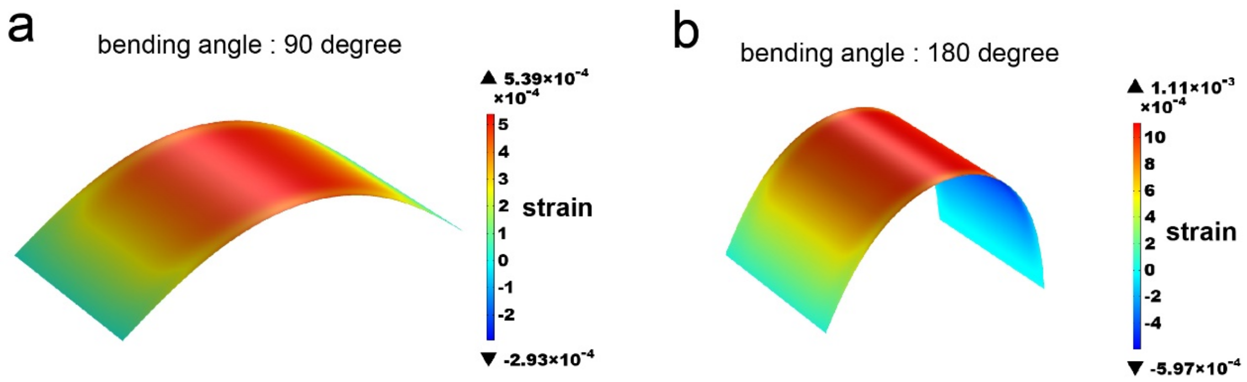

Supplementary Figure 9. a-b. COMSOL simulation diagram of strain distribution on the device. The bending angle is 90° (a) and 180° (b).

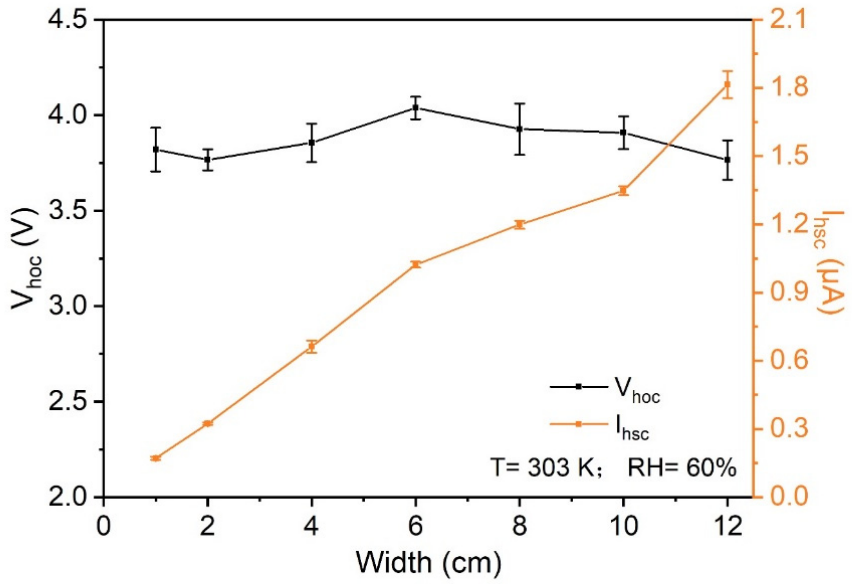

Supplementary Figure 10. Variation of open-circuit voltage and short-circuit current with width of porous *d*-Al_2O_3 hydrovoltaic generator device. The error bar is received from the V_{hoc} and I_{hsc} of 4 HGs under steady operating condition with the same width.

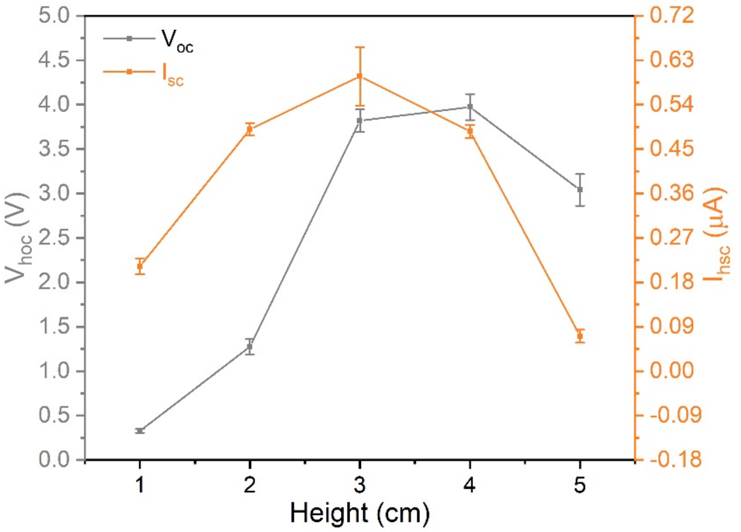

Supplementary Figure 11. Variation of open-circuit voltage and short-circuit current with heights of porous *d*-Al_2O_3 hydrovoltaic generator device. Here, the width HG devices with constant width of 4.5 cm. The ambient temperature is 295 K and the humidity is 48% RH. The error bar is received from the V_{hoc} and I_{hsc} of 4 HGs under steady operating condition with the same width.

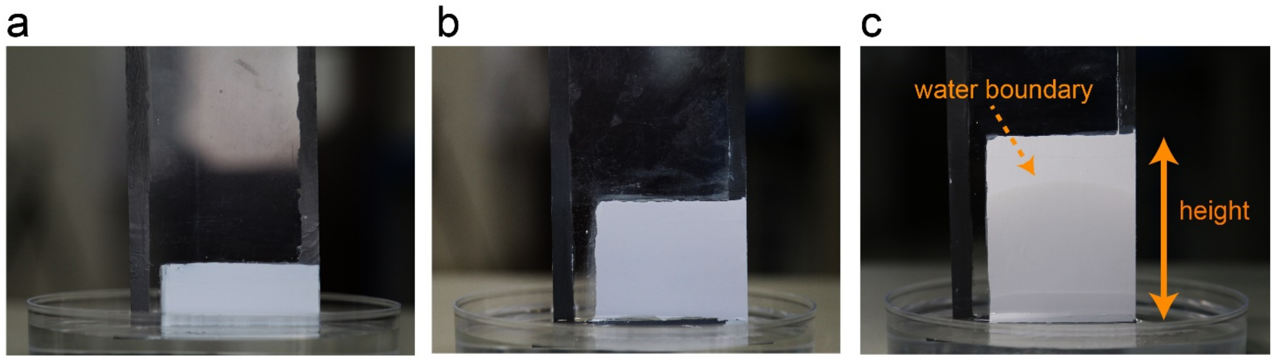

Supplementary Figure 12. a-c. Optical images showing a HG device with the height of 1, 3, and 5 cm. The ambient temperature is 295 K and the humidity is 48% RH. We fabricated a series of HG devices with constant width of 4.5 cm and different heights of 1, 2, 3, 4, and 5 cm to investigate the effect of height on the performance. Here, to express the results more clearly and conveniently, we define the open circuit voltage and short circuit current of the device at height x cm (x = 1, 2, 3, 4, and 5 cm) as $V_{oc\text{-}hx}$, and $I_{sc\text{-}hx}$, respectively. Just as the reviewer expected, when the height of the HGs increased from 1 to 4 cm, V_{oc} increased gradually from 0.33 V to 3.97 V, and $V_{oc\text{-}h3}$ is 3.82 V. However, $V_{oc\text{-}h5}$ is dramatically decreased to 3.04 V (Fig S10). Correspondingly, I_{sc} increased from 0.213 to 0.597 μA when height of the HGs increased from 1 to 3 cm. And when height of the HGs is larger than 3 cm, I_{sc} will dramatically decreased.

In order to explain the above results, we observed the distribution of water on the Al_2O_3 film. As shown in the Fig S11, water can climb to a height of 3.5 cm in the Al_2O_3 film at 48% RH, and is distributed in a gradient in the film due to gravity. When height of the HGs increased from 1 to 4 cm, the amount of water flowing through the nanochannel in the film increases correspondingly, resulting in a higher voltage. However, Al_2O_3 is a very poor conductivity material. When the height of the device is greater than 3.5 cm, the internal resistance of the device will increase sharply, resulting in a rapid decrease in the current of the device. The voltage measured is 3.04 V for the device of 5cm, which is because extra-large internal resistance can also hinder voltage testing on equipment according to the principle of voltage division.

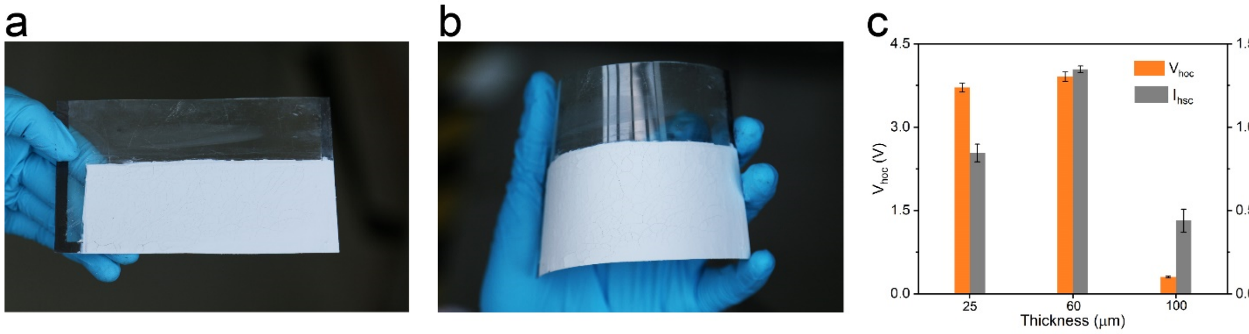

Supplementary Figure 13. a-b. Optical image showing a HG device with 100 μm Al_2O_3 layer under relaxed and bending state. **c**. V_{hoc} and I_{sc} of HGs with 25, 60, and 100 μm Al_2O_3 layer respectively**.** It was found that cracks would occur without bending in the manufacturing process for 100 μm thick films, which is because the rapid evaporation of the solvent (ethanol) will cause the shrinkage of the film, and the thick film cannot release the shrinkage stress sufficiently, resulting in cracks. When we bended the device, the spacing and number of the cracks dramatically increased.

In general, the relationship between the bending stiffness (*EI*) and thickness of materials satisfies the following equation:

$$EI = E_{Pa}bh\left(\frac{1}{3}h^2 - hy + y^2\right)$$

where E_{Pa} represents the Young's modulus, and b, h, and y represent film width, its thickness, and thickness to the natural axis respectively. Because *EI* exhibits a cubic dependence on the film thickness, the deformation of the device will cause dramatically increase of cracks. Therefore, the

mechanical stability of the 100 μm Al_2O_3 layer on flexible PET substrate is not able to meet our requirements for a stable flexible hydrovoltaic power generator.

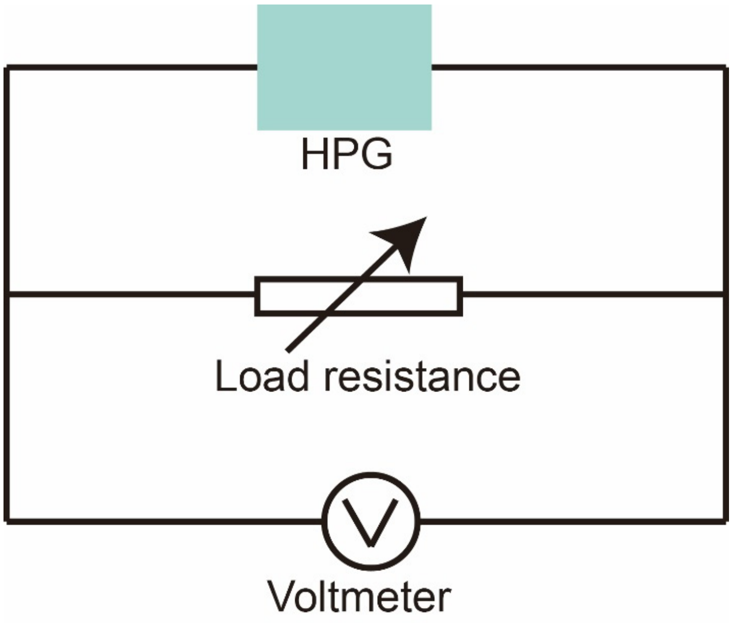

Supplementary Figure 14. Schematic illustration of output power measurement.

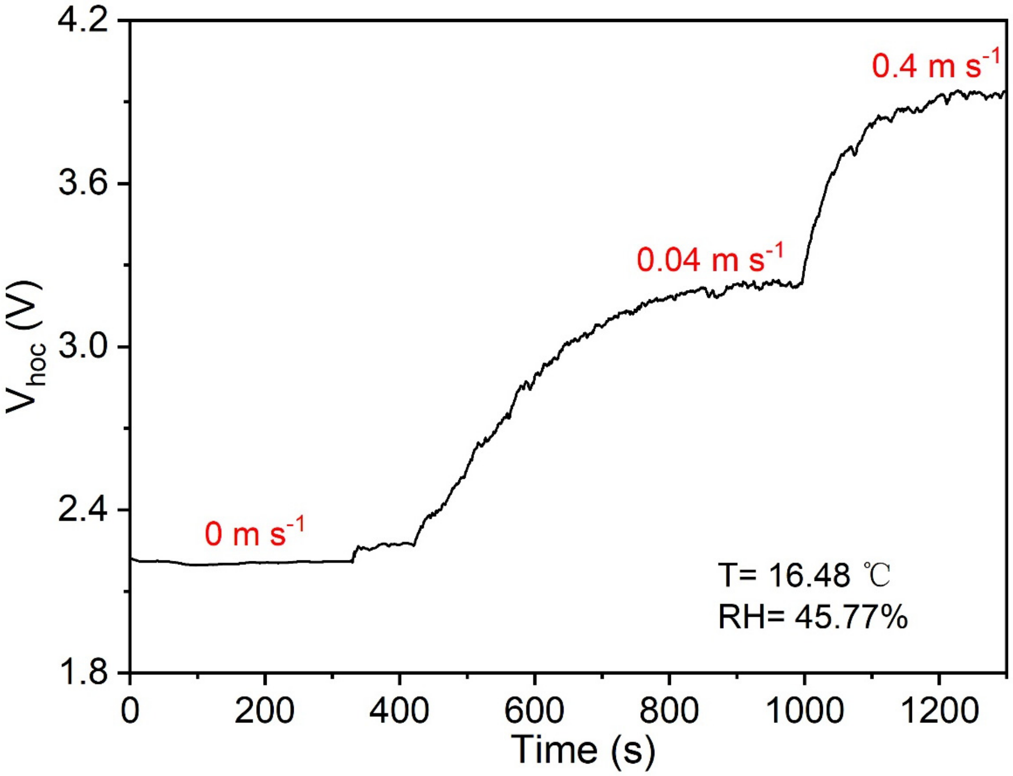

Supplementary Figure 15. Open-circuit voltage variation as a function of wind speed.

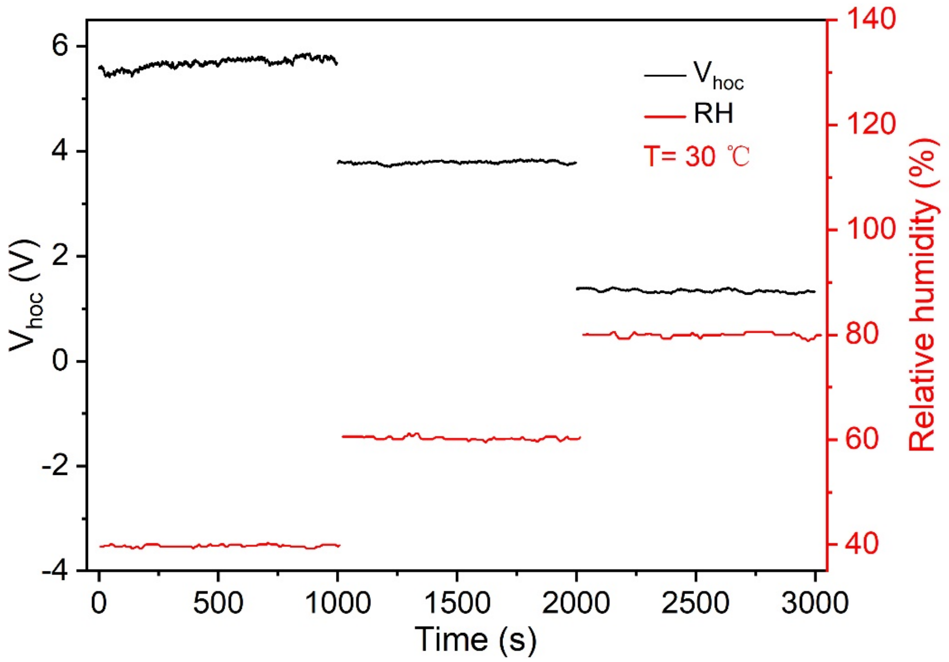

Supplementary Figure 16. V_{hoc} variation as a function of relative humidity at a stable environmental temperature of 303.2 K.

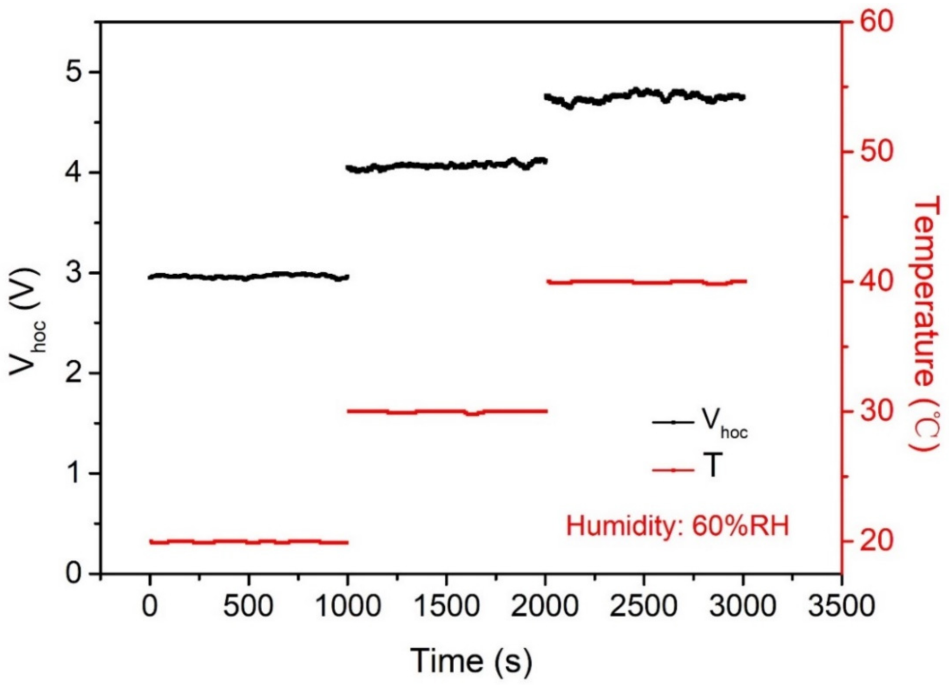

Supplementary Figure 17. V_{hoc} variation as a function of temperature at a stable relative ambient humidity of 60% RH.

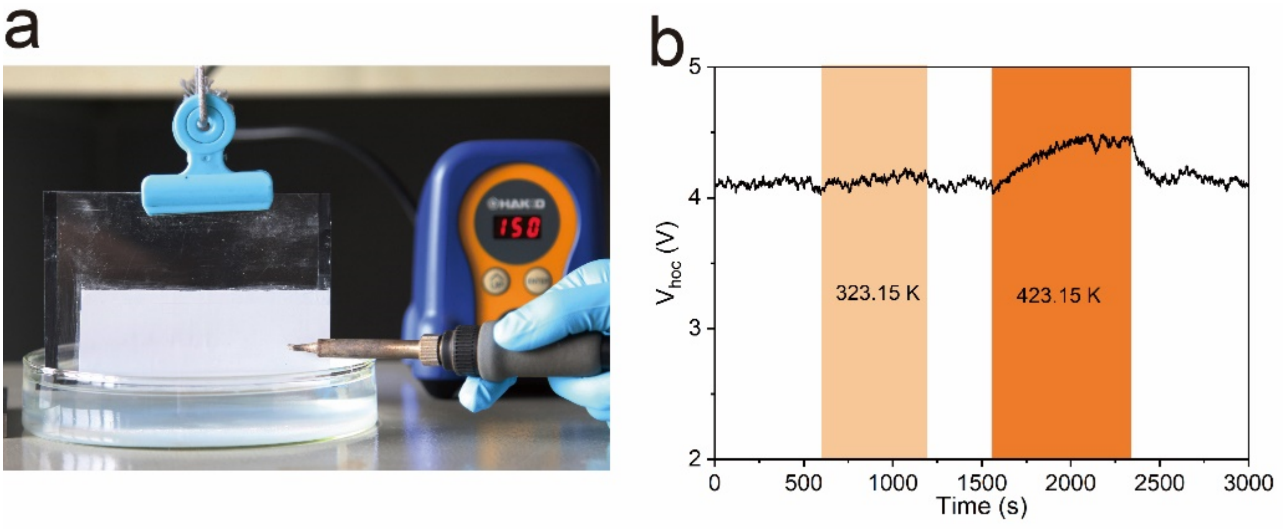

Supplementary Figure 18. a. Optical photographs show the test conditions. **b.** Variation of V_{hoc} as a function of time. We constructed the scenario by placing a heated electric iron at one end of the device to simulate extreme uneven heat distribution, as shown in the Supplementary Figure 18a. The soldering iron temperature is set at 323.15 and 423.15 K respectively. Here the room temperature is 306.15 K and relative humidity is 43 %.

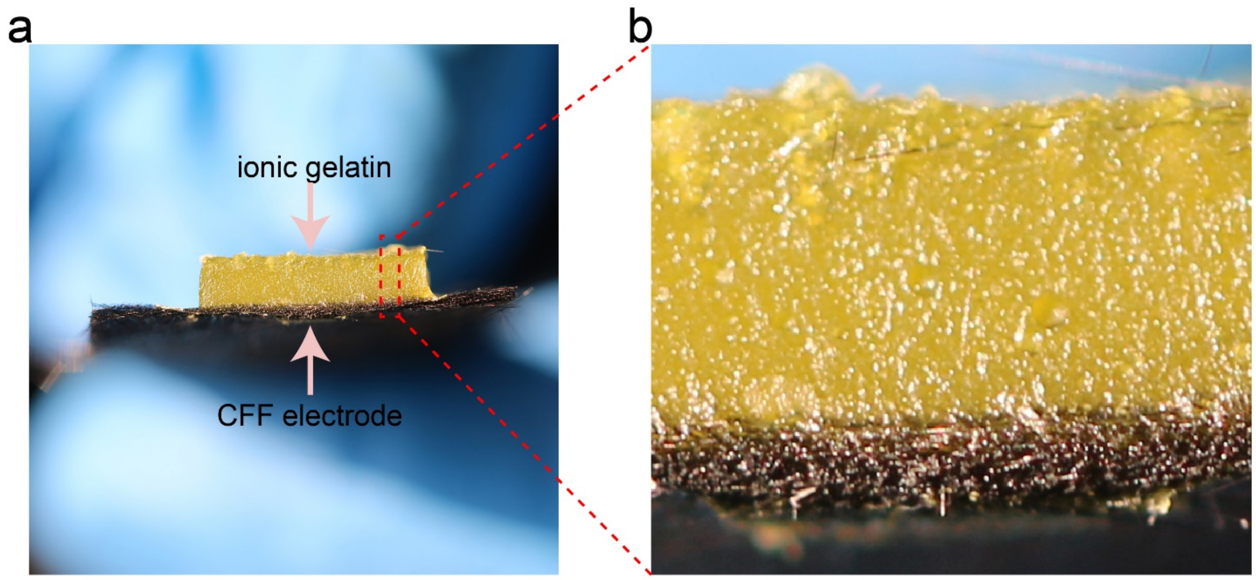

Supplementary Figure 19. a. Photograph of ionic gelatin and CFF electrode. **b**. enlarged view, proving good binding.

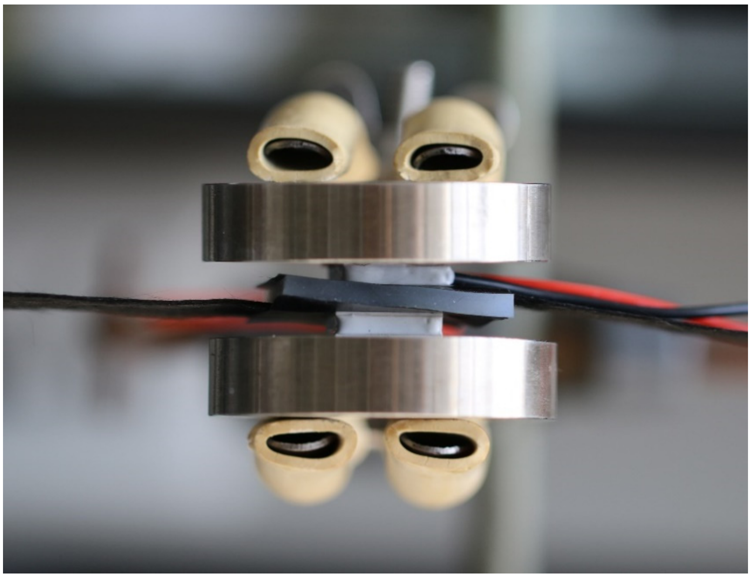

Supplementary Figure 20. Photograph of thermoelectric device measurement.

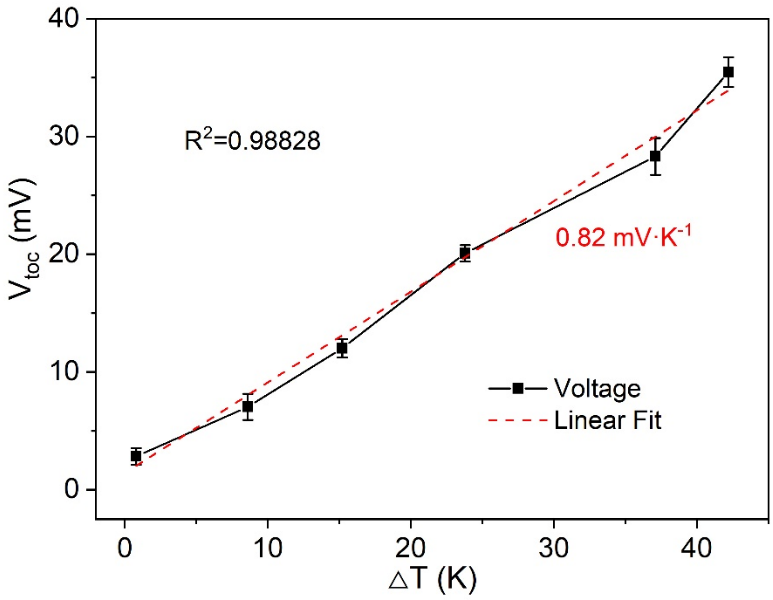

Supplementary Figure 21. Open-circuit voltage change of the TG as the function of the variation of the temperature difference. The error bar is received from the V_{toc} of 4 TGs under steady operating condition with the same ΔT.

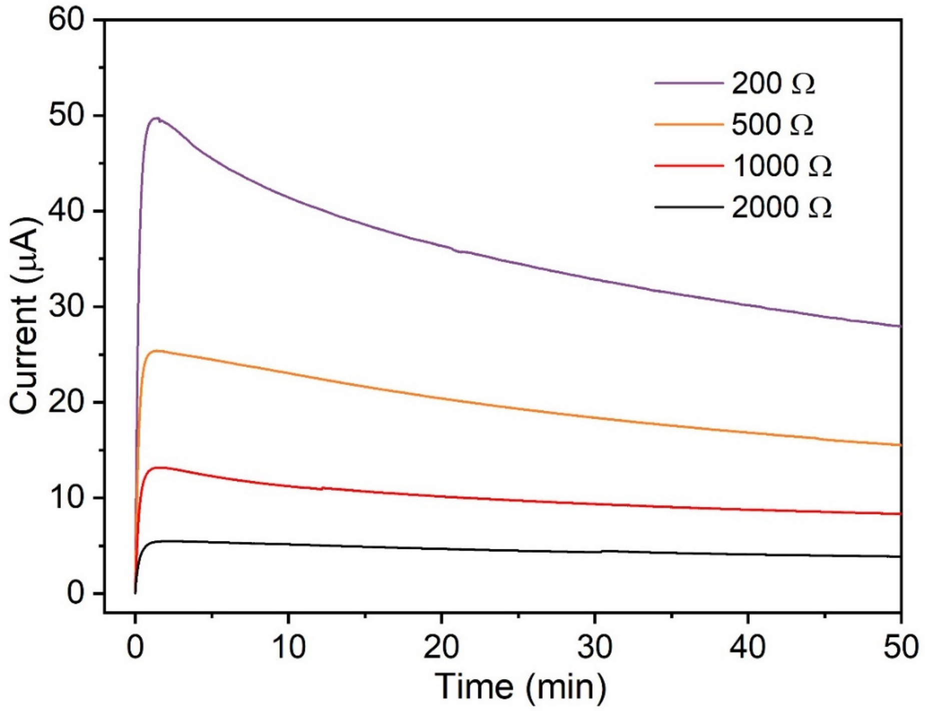

Supplementary Figure 22. Real-time current change of the as fabricated TG module with different load resistance at the ΔT of 8.6 K.

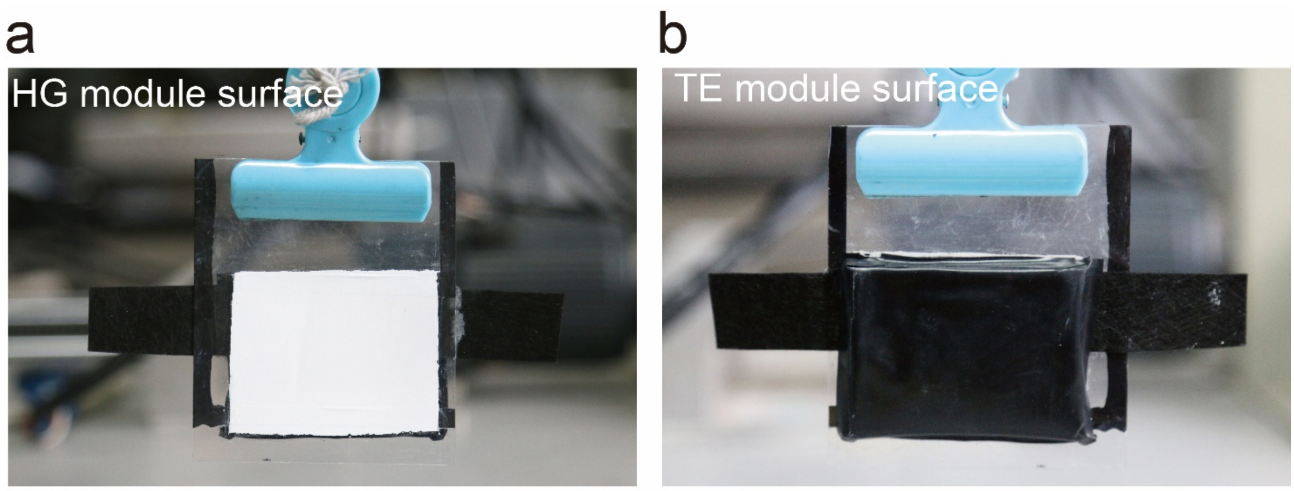

Supplementary Figure 23. a-b. Optical images showing the HG surface (a) and back side (b) HCEHG.

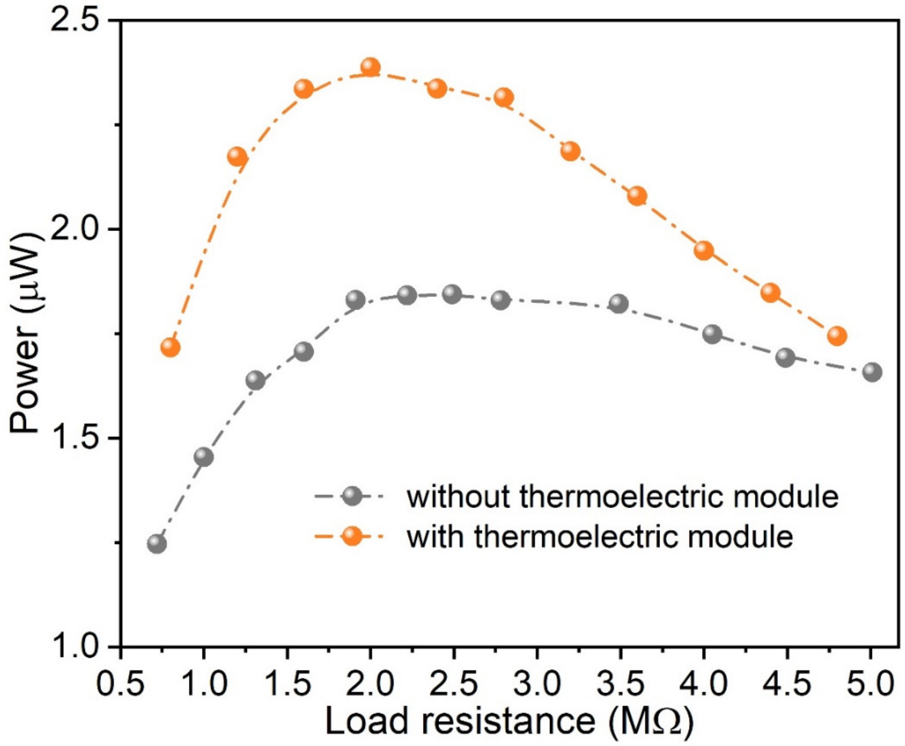

Supplementary Figure 24. Output power of the power generators with and without the thermoelectric module (size: width: 10 cm, height: 3 cm) as functions of the load resistance in a room environment.

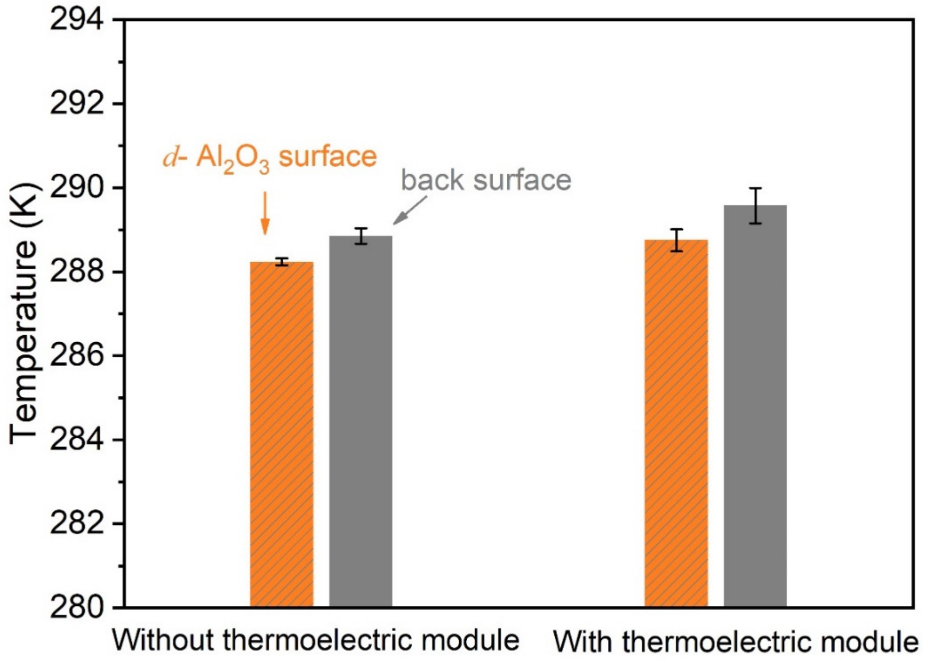

Supplementary Figure 25. Temperature of d-Al_2O_3 surface and back surface. The error bar is received from the V_{toc} of 8 devices under steady operating condition.

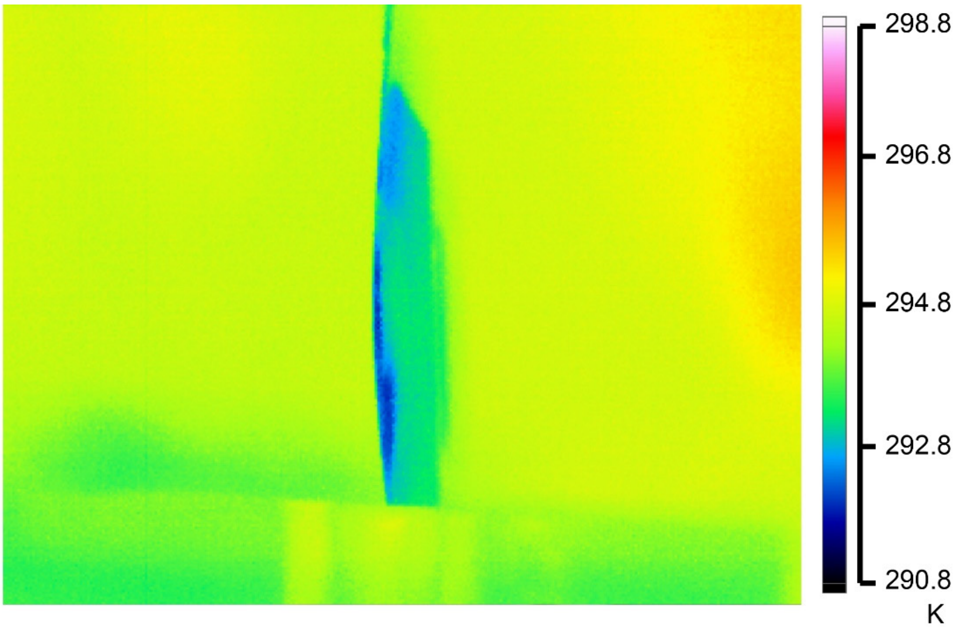

Supplementary Figure 26. Vertical infrared thermal image of the heat conduction effect enhanced hydrovoltaic power generator. The ambient temperature is 294.5 K.

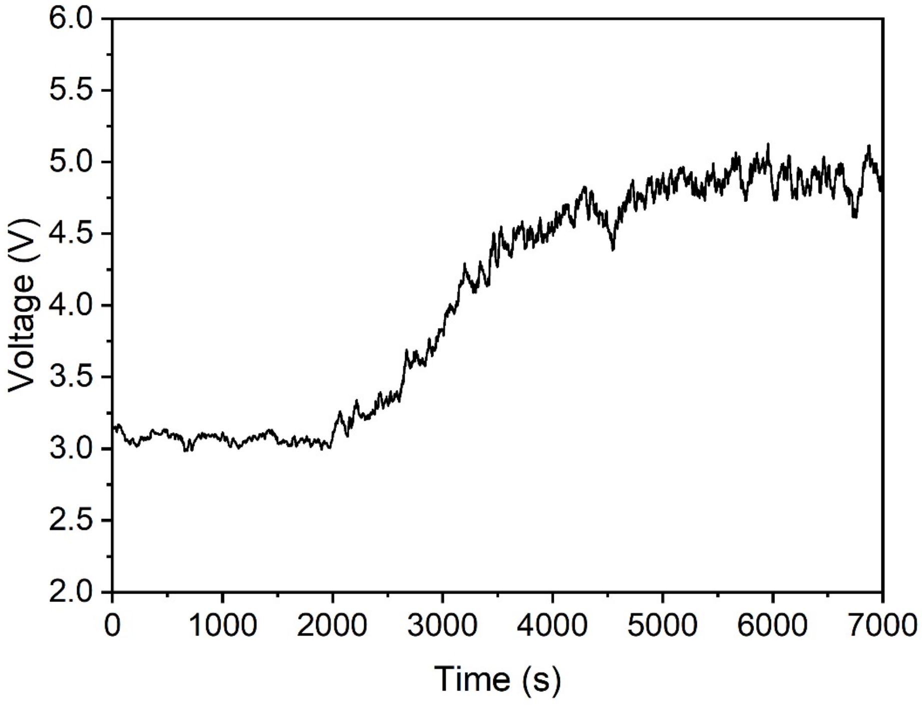

Supplementary Figure 27. Open circuit voltage response versus time curve of the hydrovoltaic device without thermoelectric module under an optical density of 1 kW m^{-2} at the ambient temperature of 295.4 K and humidity of 22.3% RH.

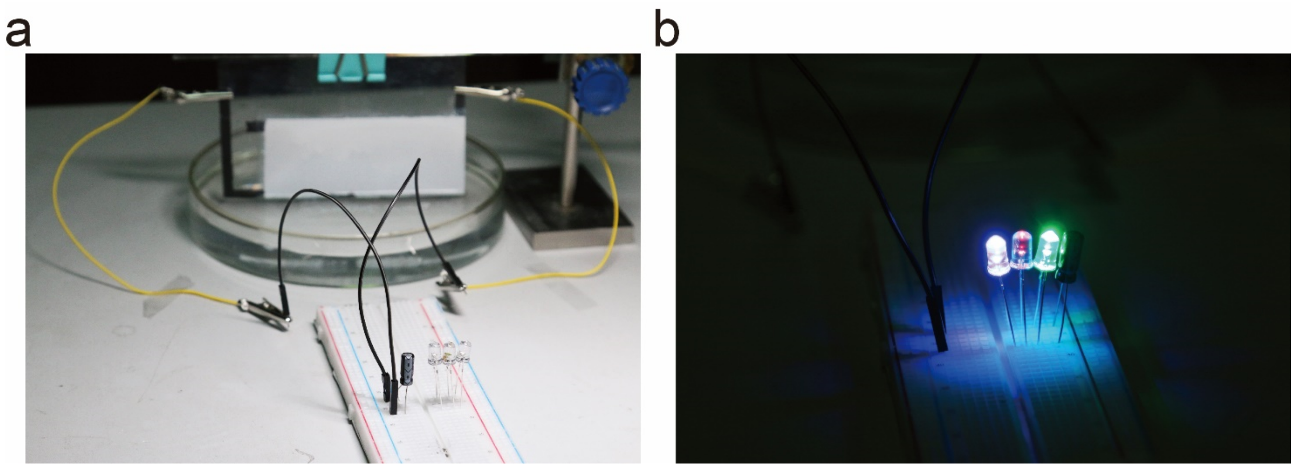

Supplementary Figure 28. Optical image showing **a**. the hydrovoltaic module (size: width: 10 cm, height: 3 cm) charging the capacitor. **b**. the capacitor powering the LEDs.

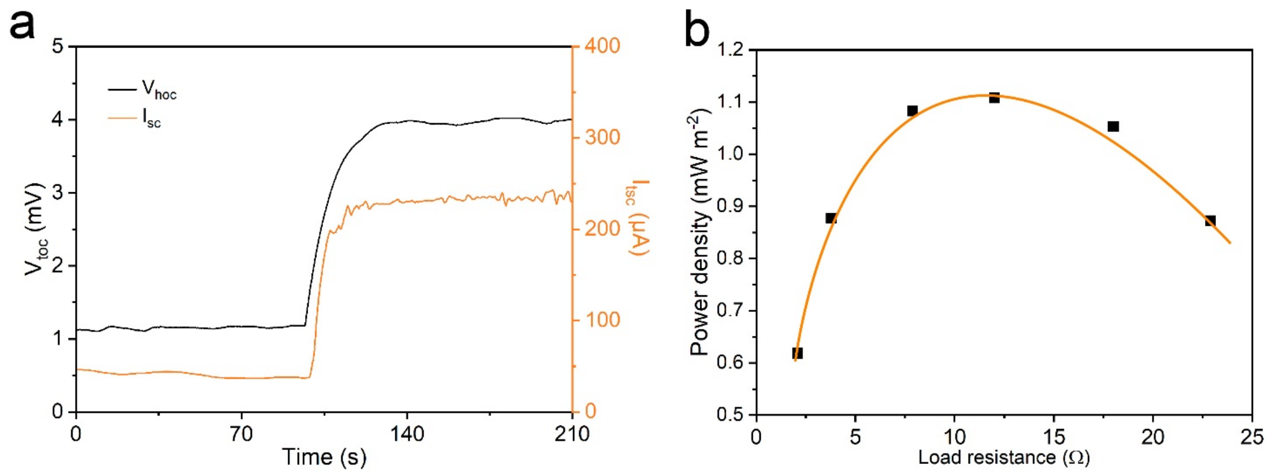

Supplementary Figure 29. a-b. the voltage, current and the power density of the generator by harvesting human body heat.

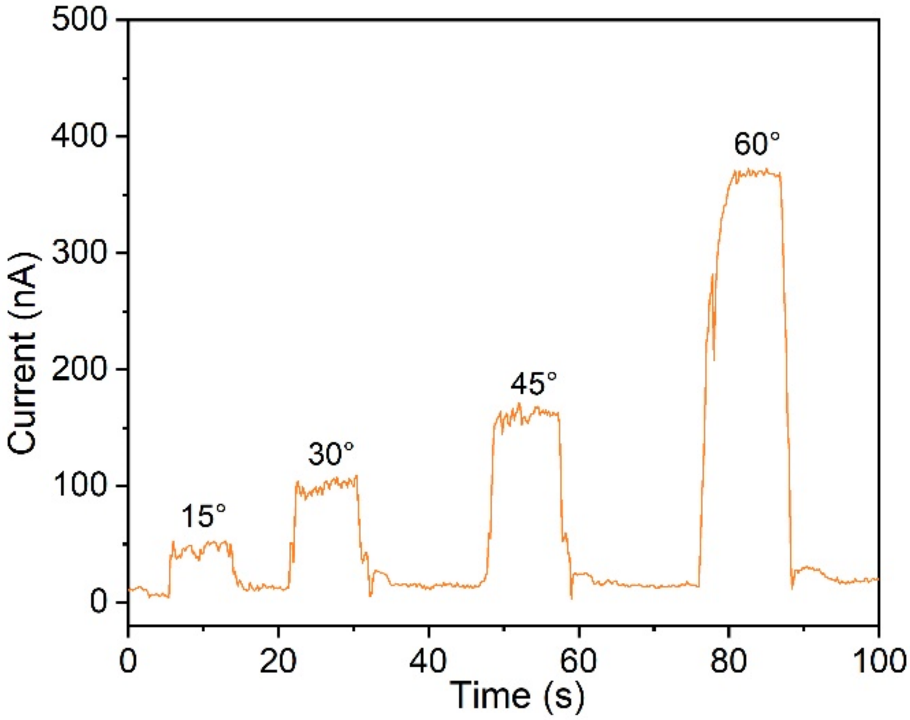

Supplementary Figure 30. Real-time current change of the CNT pressure sensor driven by thermoelectricity module showing the bending angle of neck.
